# Directed evolution and secretory expression of a pyrethroid-hydrolyzing esterase with enhanced catalytic activity and thermostability

**DOI:** 10.1186/s12934-017-0698-5

**Published:** 2017-05-11

**Authors:** Xiaolong Liu, Mingjun Liang, Yuhuan Liu, Xinjiong Fan

**Affiliations:** 1 School of Basic Medical Sciences, Anhui Medical University, 81 Meishan Rd., Hefei, 230032 Anhui People’s Republic of China; 2 School of Life Sciences, Sun Yat-sen University, 135 W. Xingang Rd., Guangzhou, 510275 Guangdong People’s Republic of China

**Keywords:** Pyrethroids, Random mutagenesis, *Pichia pastoris*, Secretory expression

## Abstract

**Background:**

Pyrethroids are potentially harmful to human health and ecosystems. It is necessary to develop some efficient strategies to degrade pyrethroid residues. Biodegradation is generally considered as a safe, efficient, and inexpensive way to eliminate environmental contaminants. To date, although several pyrethroid-hydrolyzing esterases have been cloned, there has been no report about a pyrethroid hydrolase with high hydrolytic activity, good stability, and high productivity, indispensable enzymatic properties in practical biodegradation. Almost all pyrethroid hydrolases are intracellular enzymes, which require complex extraction protocols and present issues in terms of easy inactivation and low production.

**Results:**

In this study, random mutagenesis was performed on one pyrethroid-hydrolyzing esterase, Sys410, to enhance its activity and thermostability. Two beneficial mutations, A171V and D256N, were obtained by random mutagenesis and gave rise to the mutant M2. The mutant displayed ~1.5-fold improvement in the *k*cat/*K*m value and 2.46-fold higher catalytic activity. The optimal temperature was 10 °C higher than that of the wild-type enzyme (55 °C). The half-life at 40–65 °C was 3.3–310 times longer. It was surprising that M2 has a half-life of 12 h at 70 °C while Sys410 was completely inactivated at 70 °C. In addition, the desired gene was extracellularly expressed in a *Pichia pastoris* host system. The soluble expression level reached up to 689.7 mg/L. Remarkably, the enzyme could efficiently degrade various pyrethroids at moderate temperature for 15 min, exceeding a hydrolysis rate of 98%, which is the highest value ever reported.

**Conclusions:**

This is the first report about random mutagenesis and secretory expression of pyrethroid-hydrolyzing esterase with high-level productivity and purity in *P. pastoris*. Broad substrate specificity, enhanced activity and thermostability make M2 an ideal candidate for the biodegradation of pyrethroid residues.

**Electronic supplementary material:**

The online version of this article (doi:10.1186/s12934-017-0698-5) contains supplementary material, which is available to authorized users.

## Background

Pyrethroids have been most widely used as insecticides throughout the world in agriculture, forestry, public health, and houses, as well as for protection of textiles and buildings [1, 2]. Previously considered to be of low toxicity and great selective lethality, pyrethroids have been used for more than 30 years and have accounted for almost 25% of the global pesticide market [3]. As a replacement for the more toxic and environmentally persistent organochlorine and organophosphorus pesticides, the demand for pyrethroids continues to grow [4, 5]. However, non-judicious use has raised great concerns as it has caused many problems, such as pest resistance, soil and water contamination, high residue in agricultural products, and human exposure [6, 7]. Therefore, the development of some efficient strategies to solve these problems is urgently required.

Biodegradation is generally considered as a safe, efficient, and inexpensive way of eliminating environmental contaminants [8]. The major metabolic pathway of pyrethroids involves cytochrome P450 oxidation and ester-bond hydrolysis by esterases that results in nontoxic acid and alcohol production [9, 10]. To date, many pyrethroid-degrading microorganisms have been isolated and studied [11–15]. Compared with living microorganisms, recombinant enzymes have greater potential in eliminating pyrethroid residuals, especially with mass production and safety [16]. So far, quite a few pyrethroid-degrading genes have been cloned and characterized, such as *pytY*, *estP*, *pytH*, *pye3*, *pytZ*, *sys410*, EstSt7 gene, and CMO gene [16–23]. However, no one enzyme has all the properties, such as high hydrolytic activity [22, 23], good stability [16, 23], and high productivity [17–21], which are indispensable for practical biodegradation. And almost all of them are intracellular enzymes, which require complex extraction protocols and present issues in terms of easy deactivation and low production [24]. To facilitate downstream processing in large-scale biotechnological applications, secretion of the overexpressed enzymes into the culture medium is desirable [25, 26].

Increasing interest in applying enzymes to industry has spurred the search for biocatalysts with new or improved properties [27]. Directed evolution has emerged as a ubiquitous technique to enhance the stability, activity, and selectivity of enzymes [28]. Notably, it has not relied on a priori structural and mechanistic information about the proteins [29], more suitable for modifying novel enzymes. Over the past decade, a large number of molecular biological methods for gene mutagenesis has been developed [29]. Random mutagenesis, as a powerful tool, has been advocated for modifying various enzyme functions [28, 30]. Jang et al. created a mutant β-agarase from *Zobellia galactanivorans* that had enhanced thermostability by random mutagenesis. The melting temperature (Tm) was increased by 5.2 °C over that of the wild-type enzyme (54.6 °C) [31]. Yu et al. enhanced the thermostability of a *Rhizopus chinensis* lipase by two rounds of error-prone polymerase chain reaction (PCR) and two rounds of DNA shuffling. The S4-3 variant was the most thermostable lipase; Tm was 22° higher and the half-lives at 60 and 65 °C were 46 and 23 times longer [32]. For practical use in clinical diagnosis, directed evolution was applied to improve the thermostability of fructosyl peptide oxidases. The sextuple mutant enzyme, R94K/G184D/F265L/N272D/H302R/H388Y, had a half-life of thermal inactivation at 50 °C that was 79.8-fold longer than that of the parental fructosyl peptide oxidase [33].

In our previous work [21], we identified a novel pyrethroid-hydrolyzing enzyme, Sys410, with broad substrate specificity and relatively high activity. High catalytic activity and thermostability are very attractive properties for practical applications. Herein, we chose Sys410 as a starting point for further improvement. Two beneficial mutations were obtained by random mutagenesis and gave rise to the mutant M2. The enzymatic properties and pyrethroid degradation were well characterized. Meanwhile, the desirable mutant was extracellularly expressed with high-level productivity and purity in *Pichia pastoris*.

## Results and discussion

### Directed evolution and screening for mutants with enhanced activity and stability

**Fig. 1:**
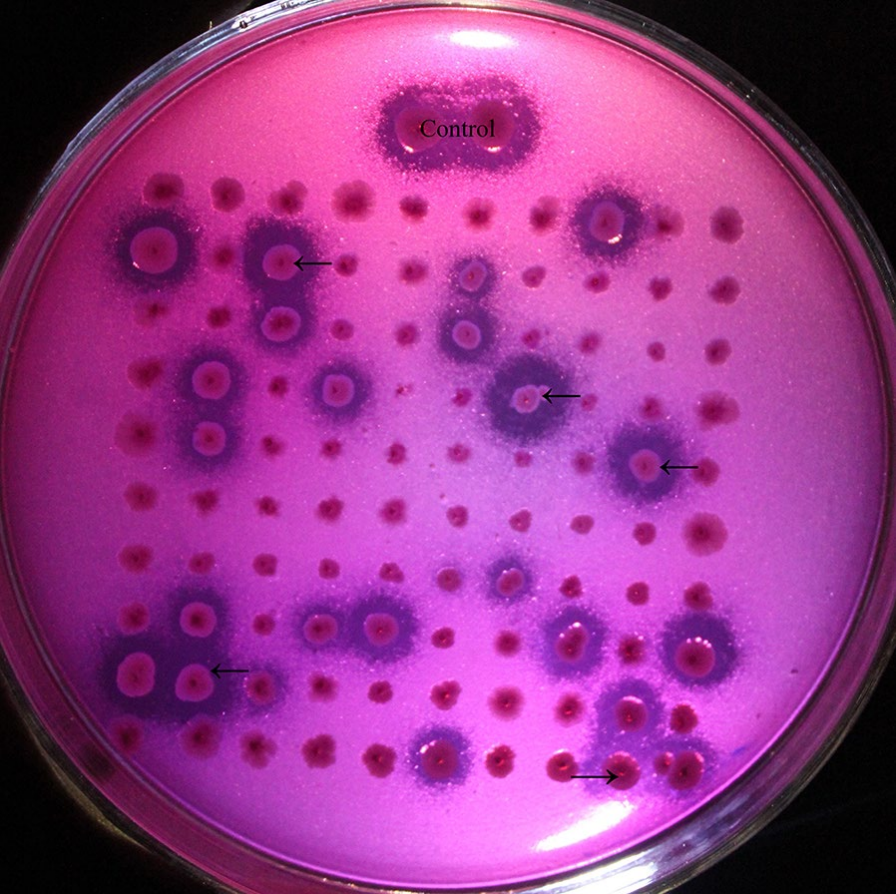
Screening of mutants with better activity (→). The transformants were replicated on LB agar plates supplemented with 0.1% (v/v) tributyrin. A transparent zone due to tributyrin hydrolysis appeared at 37 °C in 1–2 days. Clones with enhanced activity displayed bigger hydrolysis zones on tributyrin plates

**Fig. 2:**
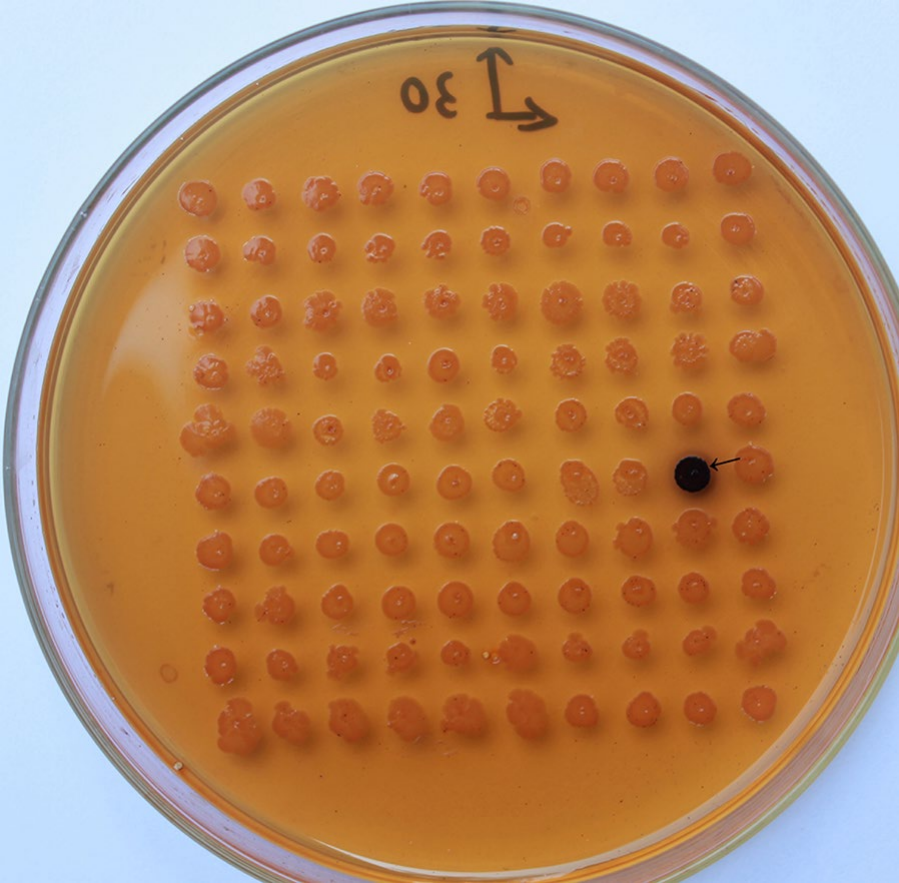
Screening of mutants with better thermostability (→). After being treated at 60 °C for 55 min, the clones were overlayed with 0.3 mg/mL α-naphthyl acetate and 1.3 mg/mL Fast Blue B. Clones with enhanced thermostability (M1) rapidly turned* deep brown* around the colony

In the study, random mutations were introduced into the *Sys410* gene by error-prone PCR. A two-step screening strategy was designed for screening. For the first round of error-prone PCR, plasmid pET28a-*Sys410* was used as the template. Several clones displayed bigger hydrolysis zones on tributyrin plates (Fig. 1), and were then evaluated for thermostability. Only one positive clone named M1 turned deep brown after being treated at 60 °C for 55 min (Fig. 2). For the second round of error-prone PCR, plasmid pTV118N-*M1* was used as the template. One positive clone harboring plasmid pTV118N-*M2* was obtained (Additional file 1: Figure S1).

A good method is crucial to screen the target gene from the enormous number of clones in a mutagenesis library. Hydrolysis areas were measured semi-quantitatively [34]. Clones with enhanced activity showed a bigger hydrolysis zone, and were selected for further study. The second screening was also based on esterase activity. In that step, it was very important to determine the critical inactivation temperature and time of the control clone harboring the template plasmid. The critical inactivation conditions were 60 °C for 55 min in the first round, and 80 °C for 60 min in the second round. After being heated, clones with enhanced thermostability still had esterase activity, and could react with α-naphthyl acetate and Fast Blue B, which resulted in a deep brown color around the colony [35], while clones with the same or reduced thermostability had no color change. In the end, two positive clones were obtained through two rounds of random mutagenesis. The results showed that this two-step screening strategy was efficient in screening esterases with enhanced activity and thermostability.

### Sequence and structure analysis

**Fig. 3:**
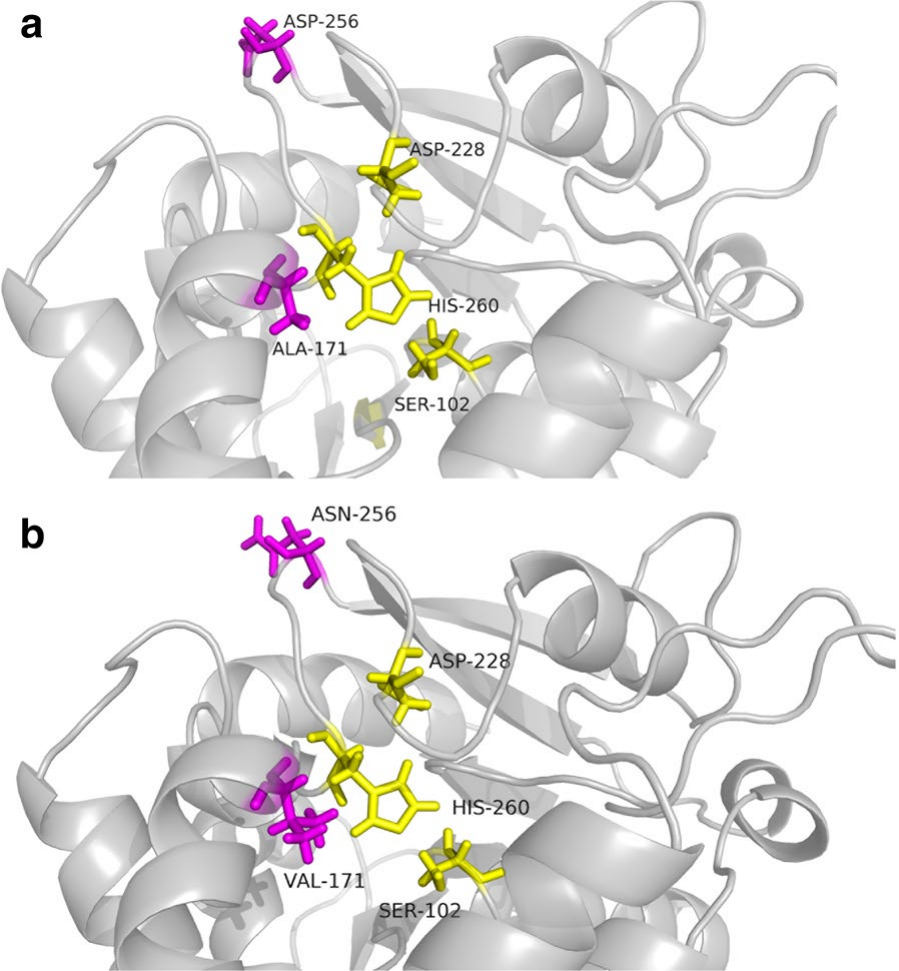
Homology models of Sys410 (**a**) and variant M2 (**b**). The catalytic triads S102-H260-D228 are colored *yellow*. Mutations are colored *carmine*

Sequence analysis identified one point mutation (C512T) in M1, and two point mutations (C512T and G766A) in M2. Nucleotide mutations at C512T and G766A caused amino acid mutations from alanine to valine at position 171 and from aspartic acid to asparagine at position 256, respectively. M3 with one point mutation (G766A) was generated by site-directed mutagenesis. Homology models of Sys410 and variant M2 were built by SWISS-MODEL (https://www.swissmodel.expasy.org) and the models were evaluated with MolProbity [36, 37] and SAVES (The Structure Analysis and Verification Server, version 4). The overall structures of the two enzymes are extremely similar (Fig. 3). The catalytic triads S102-H260-D228 are well conserved, suggesting that these enzymes share a common catalytic mechanism. Mutation A171V is inside the molecule, near to the catalytic center, and D256N is on the surface of the protein.

### Heterologous expression and purification of the mutants

The full-length mutant genes were amplified and cloned into the expression vector pET-28a (+) with a C-terminal 6 × His tag, expressed in *E. coli* BL21 (DE3) with 0.6 mM IPTG induction at 37 °C for 8 h, then purified by Ni–NTA-agarose chromatography. The sodium dodecyl sulfate–polyacrylamide gel electrophoresis analysis showed that the target recombinant protein appeared as a single band on SDS-PAGE with molecular weight 36.7 kDa (Additional file 2: Figure S2), consist of the 280 amino acids with a fusion of 54 amino acids corresponding to polyhistidine tag (His-tag), a unique thrombin cleavage site (Thrombin). The molecular weight and expression quantity of the mutants were the same as the wild-type enzyme. The fractions containing the recombinant protein were stored at −20 °C for further study.

### Determination of substrate specificity and kinetic parameters

**Fig. 4:**
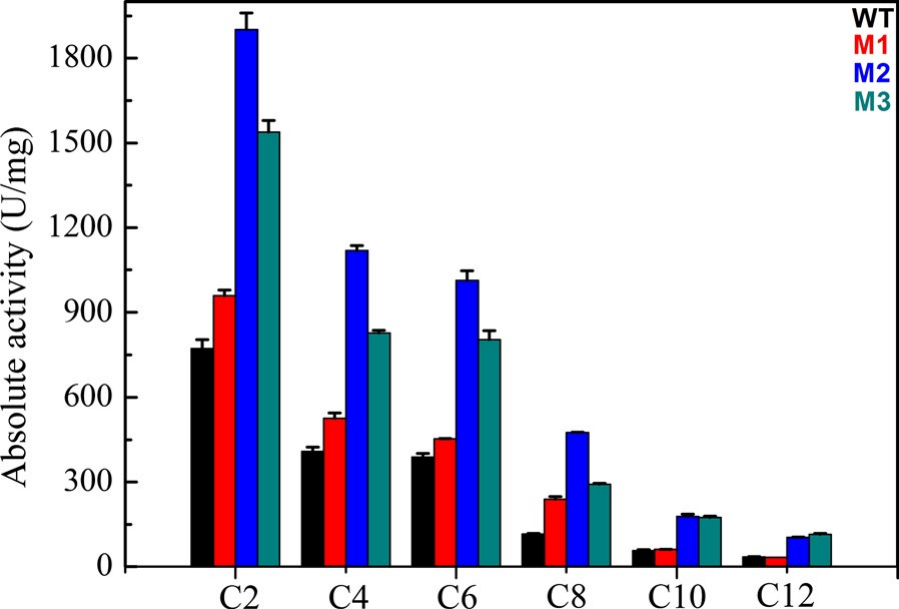
Substrate specificity of the wild-type and mutant enzymes against various ρ-nitrophenyl esters. Substrate specificity against various ρ-nitrophenyl esters with acyl chain lengths of C2, C4, C6, C8, C10, and C12 was determined under assay conditions of pH 6.5 and 55 °C. The activity of the wild-type enzyme, M1, M2, and M3 are colored *black*, *red*, *blue*, and *dark cyan*, respectively. Data points are the average of triplicate measurements, and *error bars* represent the standard deviation

To determine the substrate specificity of the mutants, we tested their activity on various ρ-nitrophenyl esters with acyl chain lengths of C2, C4, C6, C8, C10, and C12 under assay conditions of pH 6.5 and 55 °C. Substrate specificity for ρ-nitrophenyl esters of various fatty acids are shown in Fig. 4. The bar charts are very similar. All of the wild-type and mutant enzymes showed a specific preference for ρ-nitrophenyl acetate over other substrates. ρ-Nitrophenyl acetate was then used to test the activity and kinetic parameters of Sys410 and M2 (Table 1). The *K*m and *k*cat values were calculated by fitting the data to the Michaelis–Menten equation. The mutations A171V and D256N improved both the *k*cat value and the affinity. Combination of the two mutations resulted in ~1.5-fold improvement in the *k*cat/*K*m value. Generally, for most of esterases, there is a negative correlation between *K*m and *k*cat values for one enzyme toward different substrates. A low *K*m value for a substrate indicates positive affinity for the enzyme, followed with higher catalytic activity and consequently a higher *k*cat value. The catalytic activity of M2 was 2.46-fold higher than that of Sys410. The activity against ρ-nitrophenyl acetate was much better than other pyrethroid-hydrolyzing esterases reported [16–19, 21–23], except PytZ with no data shown [20]. Efficient catalytic activity is a very attractive property of enzymes for practical applications.

**Table 1:** Kinetic characterization and enzyme activity of the wild-type and mutant enzymes

Enzyme	*K*m (μM)	*k*_cat_ (s^−1^)	*k*_cat_/*K*m (s^−1^ M^−1^)	Maximum enzyme activity (U/mg)	Fold
Sys410	14.11 ± 3.22	289.12 ± 3.03	20.50	772.92 ± 3.41	1.00
M1	13.70 ± 2.02	293.31 ± 1.27	21.41	958.43 ± 1.27	1.24
M2	11.61 ± 2.74	350.15 ± 6.05	30.16	1901.31 ± 4.20	2.46
M3	12.77 ± 1.92	307.32 ± 4.89	24.07	1538.14 ± 2.21	1.99

### Effect of pH and temperature on enzyme activity

**Fig. 5:**
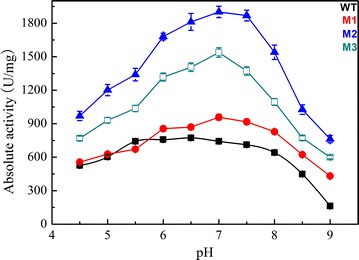
Effect of pH on the activity of the wild-type enzyme (*filled square*), M1 (*filled circle*), M2 (*filled triangle*), and M3 (*open square*). The optimum pH was measured using ρ-nitrophenyl acetate as a substrate at 55 °C. pH stability was tested after incubation of the purified enzyme for 24 h at 30 °C in the above different buffers. Data points are the average of triplicate measurements, and *error bars* represent the standard deviation

Enzyme activity can be significantly influenced by pH value and temperature. In this work, the effect of pH on the enzyme activity was determined between pH 4.5 and pH 9.0. The optimal pH was 0.5 higher than that of the wild-type enzyme (pH 7.0) (Fig. 5). Mutants have higher activity than WT over a range of pH values. Similar results were obtained in most other pyrethroid hydrolases [16–21, 23], very different from alkaliphilic EstSt7 [22]. Good pH adaptability is indispensable when dealing with the frequently changeable conditions during bioremediation.

**Fig. 6:**
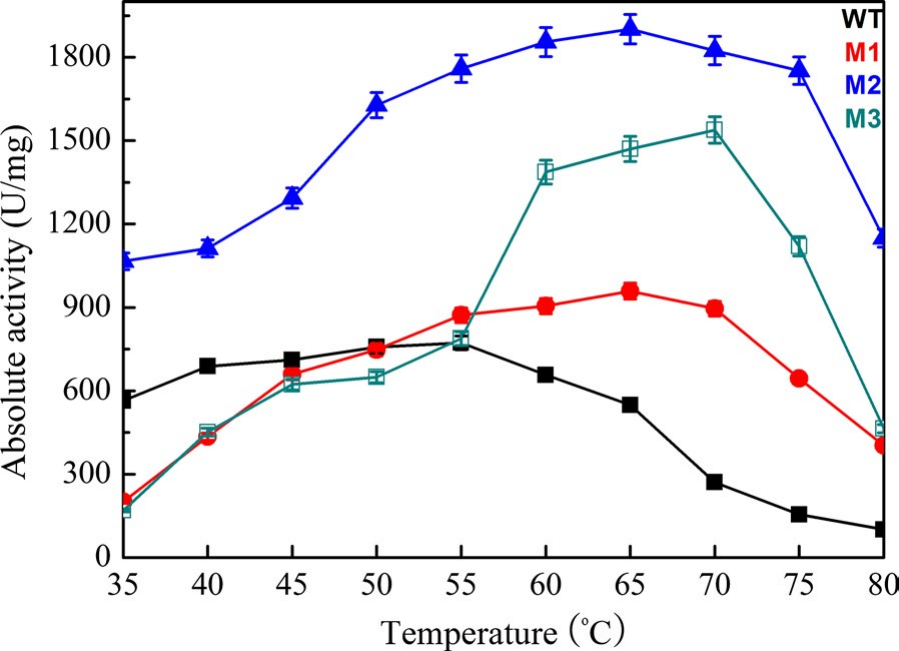
Effect of temperature on the activity of the wild-type enzyme (*filled square*), M1 (*filled circle*), M2 (*filled triangle*), and M3 (*open square*). The optimum temperature was determined analogously by measuring esterase activity in the temperature range of 35–80 °C in a phosphate buffer (50 mM, pH 6.5). Data points are the average of triplicate measurements, and *error bars* represent the standard deviation

In the present study, the activity of Sys410 and mutants was determined at temperatures of 35–80 °C (Fig. 6), with the consideration of environmental and industrial complexity. The mutants M1 and M2 had an increased optimal temperature (T_opt_) of 65 °C, 10° higher than that of the parental enzyme. The mutant M3 had an increased T_opt_ of 70 °C, 15° higher than that of Sys410. Interestingly, the relative activity of M2 was more than 50% in a wide temperature range between 35 and 80 °C, which proved that this pyrethroid-hydrolyzing enzyme possesses remarkable adaptability over a broad range of temperatures.

**Fig. 7:**
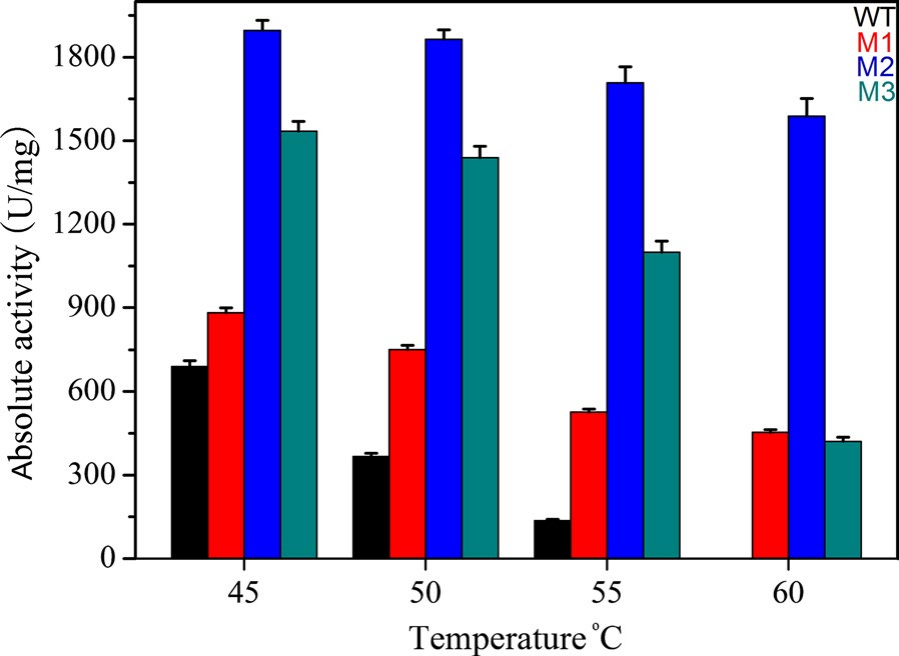
Effect of temperature on stability of the wild-type enzyme (*black*), M1 (*red*), M2 (*blue*) and M3 (*dark cyan*). Thermostability was measured by preincubation of the purified enzymes in 50 mM potassium phosphate buffer (pH 6.5) at 45–65 °C for 12 h. Data points are the average of triplicate measurements, and *error bars* represent the standard deviation

Thermostability was determined by analysis of residual activity after preincubation at 45–65 °C for 12 h (Fig. 7). Mutants exhibited excellent thermostability (Table 2; Fig. 7). After preincubation at 65 °C for 12 h, M2 still retained as much as 76.4% of its total activity. Therefore, the thermostability of M2 was further analyzed by measuring the half-lives (T_1/2_) at 40–80 °C. Table 2 lists the T_1/2_ values of wild-type (WT) and mutant M2 at different temperatures. The T_1/2_ at 40–65 °C were 3.3–310 times longer than the WT. For practical applications, enhancement of the thermostability of the targeted enzyme is generally favorable, as it will extend the shelf life of the enzyme in reagents. It was surprising that M2 had a T_1/2_ of 12 h at 70 °C whereas the WT was immediately deactivated. These results proved that M2 is highly thermostable, much better than most of the pyrethroid hydrolases aforementioned, slightly less than the thermophilic pyrethroid-hydrolyzing enzyme EstSt7. PytZ lost enzymatic activity when it was incubated at 60 °C for 2 h [20]. When the temperature was higher than 45 °C, the stability of PytY began to decrease [16]. EstP and Pye3 were both fairly stable up to 45 °C and had 54% of their activity at 50 °C, but were completely inactivated at 65 °C [17, 19]. The enzyme PytH was fairly stable up to 50 °C, had 55% residual activity at 60 °C, and was completely inactivated at 70 °C [18]. CMO was highly stable below 10 °C, but at temperatures above 10 °C, catalytic activity decreased slowly along with increased incubation time [23]. EstSt7 had an optimum temperature of 80 °C [22]. Nevertheless, it showed worse adaptability to low and moderate temperatures, which are frequently encountered during degradation of pyrethroid residues.

**Table 2:** The half-lives (T_1/2_) of Sys410 and M2 at different temperatures

Temperature (°C)	T_1/2_ of Sys410 and residual relative activity	T_1/2_ of M2 and residual relative activity	Fold improvement
40	72 h (52.6%)	236 h (50.7%)	3.3
45	38 h (50.4%)	154 h (53.8%)	4.1
50	10 h (49.8%)	133 h (54.1%)	13.3
55	6 h (56.0%)	77 h (48.3%)	12.8
60	84 min (51.4%)	53 h (54.6%)	37.9
65	6 min (48.9%)	31 h (52.8%)	310
70	–	12 h (56.1%)	–
80	–	4 h (45.3%)	–

Enzymatic properties showed that the two amino acid substitutions, A171V and D256N, were beneficial for the enhanced activity and thermostability of Sys410. V171 is one of the two positions that underwent beneficial mutations obtained from our random screen, and the original residue was an alanine. This suggests that a bulkier residue like valine may be preferred at position 171, possibly to increase the hydrophobic interactions with substrates. D256 is on the surface of the protein and it is unlikely that this residue will interact with other residues before or after the mutation, as judged from the structure. We currently do not understand the reason for the 99% activity and thermostability enhancement caused by the D256N mutation. More details regarding the mechanisms involved in the enhanced properties will be elucidated by resolving the 3D structure and rational protein design.

### Gas chromatography analysis of degradation of different pyrethroids

**Fig. 8:**
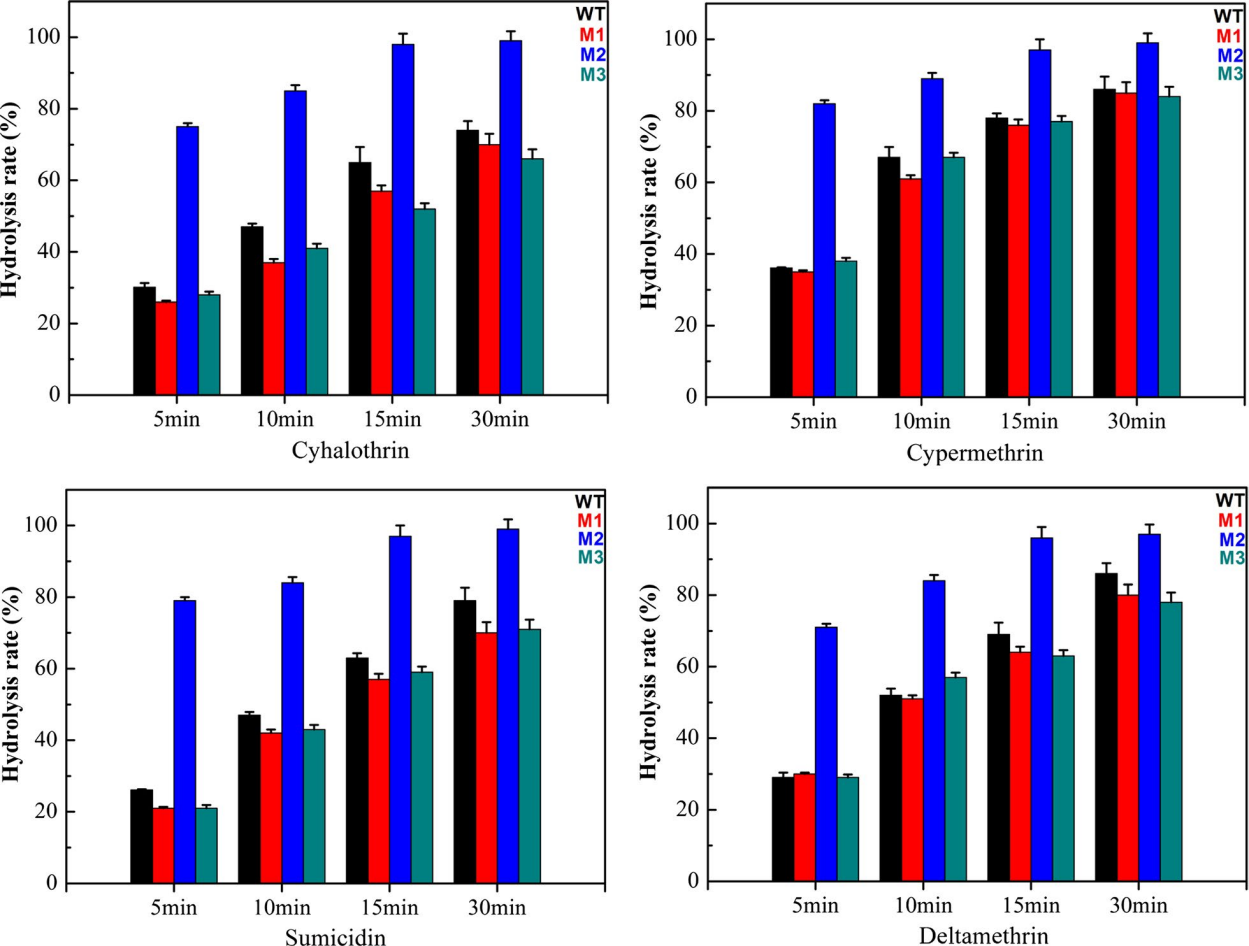
Hydrolysis rate of different pyrethroids degraded by the wild-type enzyme (*black*), M1 (*red*), M2 (*blue*), and M3 (*dark cyan*)

To assess the application potential of Sys410 and the mutants, their ability to degrade various pyrethroids was determined. Assays for pyrethroid hydrolysis were performed in 50 mM potassium phosphate buffer (pH 6.5 instead of 7.5) at 37 °C instead of high temperature for a number of reasons. Firstly, pyrethroids are unstable under alkaline conditions. Next, our aim was to evaluate the effectiveness of the enzyme at a temperature common in an application such as reducing pesticide residues in agro-products. And lastly, the hydrolysis parameters obtained could be directly compared with those of the WT Sys410 reported in the previous study. As shown in Fig. 8, the mutant M2 was able to efficiently degrade all the pyrethroids tested within a short time, indicating that M2 possessed broad substrate specificity. This feature was similar to most pyrethroid hydrolases [16–22], probably because most pyrethroid pesticides share a similar ester bond in their molecular structure. Remarkably, M2 hydrolyzed pyrethroids much more efficiently than the WT and other pyrethroid hydrolases reported [16–22], reaching over 98% conversion in 15 min. In contrast, the WT required 30 min, reaching about 80%. Unfortunately, in mid-to-low temperature conditions, both M1 and M3 had much lower activity and, as expected, the hydrolysis rates were both worse than Sys410. This demonstrated that it is very important for enzymes to possess remarkable adaptability over a broad range of temperatures to have practical application.

### Extracellular expression and characterization of M2 in *P. pastoris* X-33

Because of its remarkable enzymatic properties, the mutant M2 was selected as the candidate for further study. Secretion is preferred for heterologous protein production due to the ease of recovery [38]. The expression system of the yeast *P. pastoris* is widely used to express various proteins due to its high production and secretion efficiency [39]. Furthermore, the secreted recombinant protein in *P. pastoris* constitutes the vast majority of total proteins in the medium because the host secretes low levels of endogenous proteins [40]. In the present study, the *M2* coding sequence was cloned into the pPICZα B vector at the *Eco*RI/*Kpn*I restriction sites as described previously. This construction allowed the *M2* gene to be theoretically in-frame with the α-factor secretion signal in pPICZα B. Following a sequence check, the construct denoted as pPICZα B-*M2* was *Sac*I-linearized and electroporated into *P. pastoris* X-33. The transformant (pPICZα B-*M2*) with the highest esterase activity was selected for further experiments through activity screening and gene integration analysis.

After methanol induction for 7 days, the recombinant enzyme was secreted into culture supernatants using α-factor signal sequence and showed a single band at approximately 31 kDa by SDS-PAGE analysis (Additional file 3: Figure S3). This was in good agreement with the molecular mass deduced from the amino acid sequence with a fusion 6 × His tag, smaller than the recombinant protein in *Escherichia coli* BL21 (DE3) with a molecular weight of 36.7 kDa, consisting of 280 amino acids with a fusion of 54 amino acids corresponding to a polyhistidine tag (His tag), a unique thrombin cleavage site. It was presumed that there is little or no glycosylation in the protein. More importantly, solubility analysis revealed that the recombinant enzyme was soluble and accounted for more than 94% of the total protein in the supernatant calculated according to Quantity One software (Bio-Rad Laboratories Inc., Hercules, USA) for protein band visualization, which facilitated its purification in the application. The purity of the recombinant esterase could meet further enzyme analysis. The optimal induction time was 5 days determined by SDS-PAGE and activity analysis. The recombinant enzyme expression hit a highest level of approximately 689.7 mg/L, which was 2.87 times that expressed in *E. coli* BL21.

The expression system of the yeast *P. pastoris* is widely used for the expression of various proteins due to its high production, secretion efficiency, and few endogenous proteins in the medium [24]. However, to our knowledge, there has only been one study about a pyrethroid-metabolizing esterase expressed in *P. pastoris*. The CzEst9-pPICZα A vector transformed into *P. pastoris* GS115 produced a methanol-inducible intracellular recombinant protein, and the protein yield seemed low [41]. So, our study is the first report about extracellular and high-yield expression of a pyrethroid-hydrolyzing esterase gene in a *P. pastoris* host system.

**Fig. 9:**
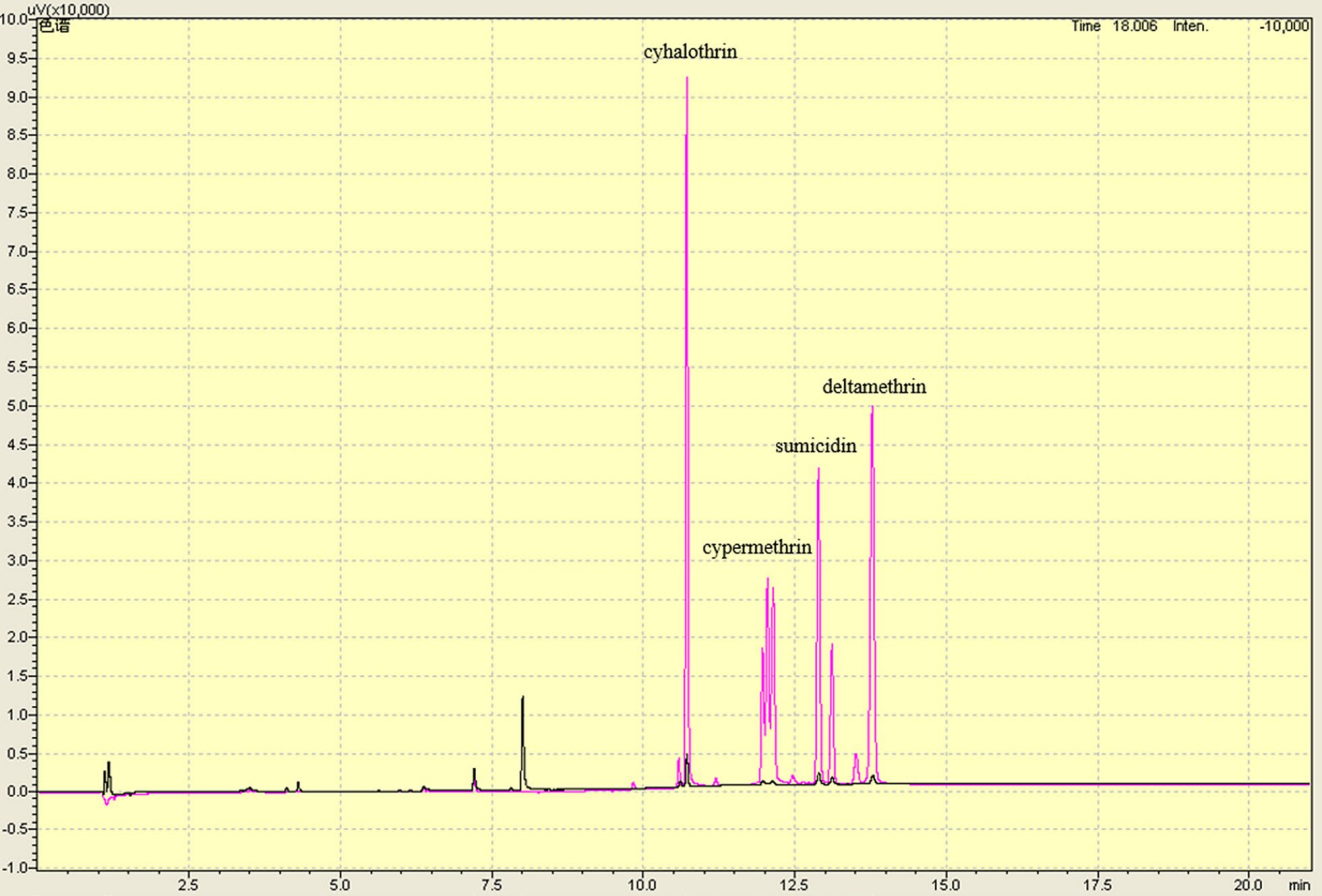
Gas chromatography analysis of different pyrethroids hydrolyzed by M2. The *red* and *black lines* denote hydrolysis chromatography by inactive enzyme (control) and active enzyme, respectively. The enzyme solution were boiled for 20 min, and then centrifugated at 13,000*g* for 10 min. The supernatants were taken as inactive enzymes

We also determined the degrading ability of M2 from the *P. pastoris* host system towards various pyrethroids. Enzyme samples (2.4 μg) with 5 mg/mL substrate in 50 mM potassium phosphate buffer (pH 6.5) were incubated at 37 °C for 15 min. As shown in Fig. 9, the hydrolysis rates of cyhalothrin, cypermethrin, sumicidin, and deltamethrin were 98.6, 99.53, 98.1, and 98.9%, respectively. The results demonstrated that the ability of the enzyme from *P. pastoris* to degrade various pyrethroids was in good agreement with that expressed in *E. coli* BL21 (DE3). Generally, pesticide residues are mixtures in the environment. A broad-spectrum pyrethroid-degrading enzyme with higher hydrolytic activity will play a better role in practical biodegradation.

## Conclusions

Pyrethroids are potentially harmful to human health and ecosystems. Thus, great concerns have been raised about pyrethroid residues and their persistence in the environment. In this study, we enhanced the activity and thermostability of one novel pyrethroid-hydrolyzing esterase by random and site-directed mutagenesis, and obtained three positive mutants. Remarkably, combination of two mutations A171V and D256N resulted in ~1.5-fold improvement in the *k*cat/*K*m value and 2.46-fold higher catalytic activity compared to the WT Sys410. In addition, the desired gene *M2* was extracellularly expressed with high yield and good solubility in a *P. pastoris* host system for the first time. More interestingly, the enzyme could efficiently degrade cyhalothrin, cypermethrin, sumicidin, and deltamethrin under assay conditions of 37 °C for 15 min, exceeding a hydrolysis rate of 98%, higher than other pyrethroid-hydrolyzing enzymes reported so far. These favorable features make M2 an ideal candidate for the biodegradation of pyrethroids. More details regarding the mechanisms involved in the enhanced properties of M2 will be elucidated by resolving the 3D structure and rational protein design; these studies are currently in progress.

## Methods

### Chemicals and materials

Technical-grade pyrethroids were provided by Jiangsu Yangnong Chemical Group Co., Ltd., Jiangsu, China. All ρ-nitrophenyl esters were purchased from Sigma. Restriction endonuclease, TaKaRa MutanBEST Kit, T4 DNA ligase and PrimeSTAR^®^ HS DNA Polymerase were purchased from TaKaRa (Dalian, China) and used according to the recommendations of the manufacturer. E.Z.N.A. Plasmid Mini Kit, E.Z.N.A. Gel Extraction Kit and E.Z.N.A. Yeast DNA Kit were purchased from OMEGA (Norcross, USA). GeneMorph^®^ II Random Mutagenesis Kit was purchased from Stratagene (La Jolla, CA, USA). Zeocin™ and EasySelect™ *Pichia* Expression Kit were purchased from Invitrogen (Carlsbad, CA, USA). All other chemicals and reagents were of analytical grade and were purchased from commercial sources, unless otherwise stated.

### Bacterial strains and plasmids

*Escherichia coli* DH5α (Novagen, Madison, WI, USA), *E. coli* BL21 (DE3) (Novagen, Madison, WI, USA) and *P. pastoris* strain X-33 (Invitrogen, Carlsbad, CA, USA) were used as the hosts for gene cloning and protein expression, respectively. pTV118N (TaKaRa, Dalian, China) and pPICZα B (Invitrogen, Carlsbad, CA, USA) were used to construct mutant libraries and express the target protein, respectively.

### Directed evolution and screening for mutants with enhanced activity and thermostability

Random mutagenesis of the *Sys410* gene (GenBank accession number: JQ272178) was carried out by error-prone PCR using a GeneMorph^®^ II Random Mutagenesis Kit at low frequency (0–4.5 mutations/kb) according to the manufacturer’s protocol, performed with pET28a-*Sys410* as a template. The following primers were used: fw (5′-TTATTGGATCCATGTTCGCTCAGCCCCCGAA-3′; the *Bam*HI cutting site is underlined) and rv (5′-CCGGAATTCTCACTCCGCCAAGAACCGATCCACG-3′; the *Eco*RI cutting site is underlined). The PCR product digested with *Bam*HI/*Eco*RI was ligated into *Bam*HI/*Eco*RI-digested cloning vector pTV118N, and then transformed into *E. coli* DH5α cells. The transformants were plated onto LB agar containing 100 μg/mL ampicillin and further incubated at 37 °C overnight.

The mutant library was screened by a two-step screening strategy. Initially, the transformants were replicated on LB agar plates supplemented with 0.1% (v/v) tributyrin, 100 μg/mL ampicillin and 0.1 mM IPTG (isopropyl-β-d-thiogalactoside) [42]. A transparent zone due to tributyrin hydrolysis appeared at 37 °C in 1–2 days. Positive clones would develop bigger transparent zones around the colony than WT clones. Then the positive clones obtained by the initial screening were transferred to LB agar plates containing 100 μg/mL ampicillin and 0.1 mM IPTG, and further incubated at 37 °C for 12 h. After heat treatment in critical inactivation conditions, the clones were overlayed with 0.3 mg/mL α-naphthyl acetate and 1.3 mg/mL Fast Blue B [35]. The positive clones rapidly turned deep brown around the colony. The positive clones from the first round of random mutagenesis were used as the template for the second round of mutagenesis. The critical inactivation conditions were 60 °C for 55 min in the first round, and 80 °C for 60 min in the second round. The detailed methods were performed as described above.

### Site-directed mutagenesis

We created mutant M3 with a single mutation by site-directed mutagenesis for determining the effect of each mutation. In vitro site-directed mutagenesis was performed by using a TaKaRa MutanBEST Kit (TaKaRa, Dalian, China) following the instructions of the manufacturer. The plasmid pET28a-*Sys410* was used as the template. The following primers were used: fw (5′-TCTGGAGCGTGTCAACGACAGTCGC-3′; the mutation site is underlined) and rv (5′-TGGACGCCCGGCAAGGTGGCGTATT-3′). The correctness of the mutant was confirmed by DNA sequencing.

### Cloning, expression, and purification of the mutants

The mutant genes were amplified by PCR with pTV118N-*gene* as a template using the primers mentioned above which contained restriction enzyme sites *Bam*HI and *Eco*RI. Amplified DNA was digested by *Bam*HI/*Eco*RI, ligated into pET-28a (+) which was linearized by *Bam*HI/*Eco*RI, then transformed into *E. coli* BL21 (DE3) cells. *E. coli* cell transformants were plated onto LB agar containing 50 μg/mL kanamycin. Transformed cells were grown in a 250 mL flask containing 50 mL of LB (50 μg/mL kanamycin) at 37 °C until the cell concentration reached an OD_600_ of 1.0, then induced with 0.6 mM IPTG. After incubation at 37 °C for 8 h with shaking at 220 rpm, cells were harvested by centrifugation (6000*g*, 10 min) at 4 °C and suspended in binding buffer (0.5 M NaCl, 5 mM imidazole, 20 mM Tris–HCl, pH 7.9). The cells were disrupted by sonication, and the supernatant was collected by centrifugation (13,000*g*, 10 min) at 4 °C. The sample was loaded onto an Ni–NTA His·Bind column pre-equilibrated with binding buffer. Then the column was washed with binding buffer and washing buffer (0.5 M NaCl, 60 mM imidazole, 20 mM Tris–HCl, pH 7.9). Finally, the bound protein was eluted with eluting buffer (1 M imidazole, 0.5 M NaCl, 20 mM Tris–HCl, pH 7.9). The fractions containing the recombinant protein were collected and stored at −20 °C.

### Determination of substrate specificity and kinetic parameters

ρ-Nitrophenyl esters are general substrates of esterases. Substrate specificity against various ρ-nitrophenyl esters with acyl chain lengths of C2, C4, C6, C8, C10, and C12 was determined under assay conditions of pH 6.5 and 55 °C, according to the method of Fan et al. [21]. The activity was tested under conditions of pH 6.5 and 55 °C (the best conditions of Sys410), pH 6.5 and 65 °C (the best conditions of M1 and M2), and pH 6.5 and 70 °C (the best conditions of M3), respectively. One unit of enzyme activity was defined as the amount of enzyme that produced 1 μmol of ρ-nitrophenol per minute under these conditions. The purified enzyme was incubated with various concentrations of ρ-nitrophenyl acetate. The final concentration ranged from 1.0 to 10.0 mM in potassium phosphate buffer (pH to 6.5). The kinetic constants were calculated by fitting the initial rate data into the Michaelis–Menten equation using GraFit software version 6 (Erithacus Software Ltd., Horley, UK).

### Effect of temperature and pH on enzyme activity

The effect of temperature and pH on the initial reaction rates of Sys410 and the mutants was determined by using ρ-nitrophenyl acetate as a substrate. The optimum pH was measured using ρ-nitrophenyl acetate as a substrate at 55 °C. The pH buffers included citric acid-NaOH buffer (pH 3.5–5.5), potassium phosphate buffer (pH 5.0–7.0), and Tris–HCl buffer (pH 6.5–9.0). pH stability was tested after incubation of the purified enzyme for 24 h at 30 °C in the above different buffers. The optimum temperature was determined analogously by measuring esterase activity in the temperature range of 35–80 °C in a phosphate buffer (50 mM, pH 6.5). Thermostability was measured by preincubation of the purified enzyme in 50 mM potassium phosphate buffer (pH 6.5) at 45–65 °C for 12 h. The residual activity was tested under conditions of pH 6.5 and 55 °C (the best conditions of Sys410), pH 6.5 and 65 °C (the best conditions of M1 and M2), and pH 6.5 and 70 °C (the best conditions of M3), respectively. In addition, the T_1/2_ of Sys410 and M2 at 40–80 °C were measured. T_1/2_ is defined as the incubation time inactivating 50% of the initial enzyme activity. Samples containing 0.1 mg/mL purified enzyme (50 mM phosphate buffer, pH 6.5) were treated by incubating for various time intervals at different temperatures. Then the residual activities were quantified by using ρ-nitrophenyl acetate as a substrate based on the method of Fan et al. [21].

### Gas chromatography analysis of the degradation of different pyrethroids

Sys410 and the mutants were tested for hydrolysis of cyhalothrin, cypermethrin, sumicidin, and deltamethrin. Hydrolytic activity of the enzyme was determined based on the method described previously [21], with slight modification. The enzyme samples (2.4 μg, a tenth of the amount in the previous study) with 5 mg/mL substrate in 50 mM potassium phosphate buffer (pH 6.5) were incubated at 37 °C at different time intervals. Then the residual pyrethroids were quantified by gas chromatography. Aliquots (1 μL) of the reaction mixtures were loaded onto a Gas chromatography system (GC-2010, Shimadzu Corporation, Japan) with ECD Detector using RestekRTX-5 column (30 m × 0.25 mm × 0.25 μm). The column flow was 2 mL/min. The column was set at 150 °C for 1 min, and heated to 270 °C at 30 °C/min^−1^. The temperature of ECD Detector was 300 °C. The electric current was 1 nA. In each measurement, the effect of nonenzymatic hydrolysis of substrates was taken into consideration and subtracted from the value measured when the enzyme was added.

### Extracellular expression of the mutant in *P. pastoris* X-33

The mutant gene *M2* was amplified by PCR with pTV118N-*Sys410*-*M5* as a template using primers which contained restriction enzyme sites *Eco*RI and *Kpn*I. The following primers were used: fw (5′-CCGGAATTCATGTTCGCTCAGCCCCCGAA-3′; the *Eco*RI cutting site is underlined) and rv (5′-ATATAGGTACCTCACTCCGCCAAGAACCGATCCACG-3′; the *Kpn*I cutting site is underlined). The PCR product was cloned into *Eco*RI- and *Kpn*I-digested pPICZα B. After being transformed into *E. coli* DH5a, some recombinant clones were selected on low salt LB agar plates containing 25 μg/mL Zeocin^™^. The proper insert orientation was checked by restriction analysis and sequencing. The recombinant plasmid was linearized with *Sac*I and electroporated into *P. pastoris* X-33. The construction of recombinant transformants was based on the method described previously [43].

Transformants were induced on BMMY plates and screened on the basis of esterase activity. Positive clones displayed a blue color by hydrolysis with 100 μM 5-bromo-4-chloro-3-indolyl caprylate, and were further tested to confirm gene integration [44]. Some transformants were selected and cultivated in liquid media in Erlenmeyer flasks, to check their production levels [45]. The best transformant was subjected to high cell-density fermentation, according to the EasySelect™ *Pichia* Expression Kit manual. Methanol was added into the culture to a final concentration of 0.5% for maintaining induction every day. At different times (day1–day7), 1 mL of the culture was centrifuged and the amount of target protein in the supernatant was estimated by activity measurement assays and SDS-PAGE. The recombinant enzyme was also tested for hydrolysis of cyhalothrin, cypermethrin, sumicidin, and deltamethrin, based on the method described above.

## Additional files

**Additional file 1.** Screening of mutants with better activity (A) and better thermostability (B) in the second round of random mutagenesis. The transformants were replicated on LB agar plates supplemented with 0.1% (v/v) tributyrin. A transparent zone due to tributyrin hydrolysis appeared at 37 °C in 1–2 days. Clones with enhanced activity displayed bigger hydrolysis zones on tributyrin plates. After being treated at 80 °C for 60 min, the clones were overlayed with 0.3 mg/mL α-naphthyl acetate and 1.3 mg/mL Fast Blue B. Clones with enhanced thermostability (M2) rapidly turned deep brown around the colony.

**Additional file 2.** SDS-PAGE of gene expression in *E. coli* BL21 (DE3). M, protein MW marker (low); lanes 1–4, supernatant of *E. coli* BL21 (DE3) (pET-28a (+)-WT), *E. coli* BL21 (DE3) (pET-28a (+)-M1), *E. coli* BL21 (DE3) (pET-28a (+)-M2), *E. coli* BL21 (DE3) (pET-28a (+)-M3); lanes 5–8, purified WT, M1, M2, M3.

**Additional file 3.** SDS-PAGE of gene expression in *P. pastoris* X-33 (pPICZα B-*M2*). M, protein MW marker (low); lanes 1–7, supernatant of *P. pastoris* X-33 (pPICZα B-*M5*) induced for 1–7 days; lane 8, supernatant of *P. pastoris* X-33(pPICZα B) at day 7; lane 9, cell lysis solution of *E. coli* BL21 (DE3) (pET-28a(+)-*M2*) (arrow). After after being treated at 60 °C for 55 min, the clones were overlayed with 0.3 mg/mL α-naphthyl acetate and 1.3 mg/mL Fast Blue B. Clones with enhanced thermostability rapidly turned deep brown around the colony.
