# Supplementary figures and images for: Directed evolution and secretory expression of a pyrethroid-hydrolyzing esterase with enhanced catalytic activity and thermostability
Source: Microb Cell Fact. 2017 May 11;16:81. doi: 10.1186/s12934-017-0698-5 (PMC5425977; doi:10.1186/s12934-017-0698-5)

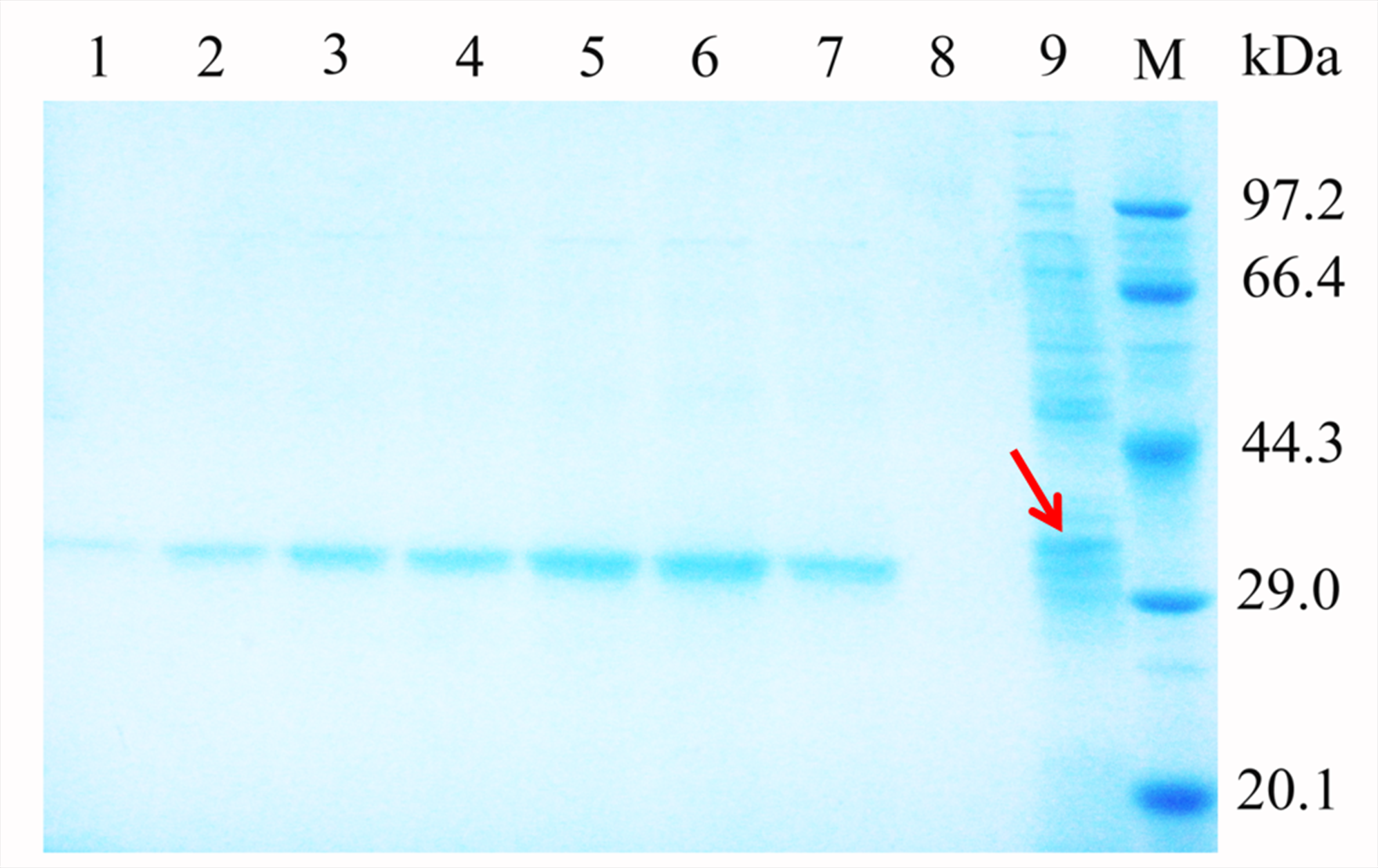

Supplement: Supplementary file 3 — Additional file 3. SDS-PAGE of gene expression in P. pastoris X-33 (pPICZα B-M2). M, protein MW marker (low); lanes 1–7, supernatant of P. pastoris X-33 (pPICZα B-M5) induced for 1–7 days; lane 8, supernatant of P. pastoris X-33(pPICZα B) at day 7; lane 9, cell lysis solution of E. coli BL21 (DE3) (pET-28a(+)-M2) (arrow). After after being treated at 60 °C for 55 min, the clones were overlayed with 0.3 mg/mL α-naphthyl acetate and 1.3 mg/mL Fast Blue B. Clones with enhanced thermostability rapidly turned deep brown around the colony. [file 12934_2017_698_MOESM3_ESM.tif]
